# Functional Characterization of Sticholysin I and W111C Mutant Reveals the Sequence of the Actinoporin’s Pore Assembly

**DOI:** 10.1371/journal.pone.0110824

**Published:** 2014-10-28

**Authors:** Valeria Antonini, Victor Pérez-Barzaga, Silvia Bampi, David Pentón, Diana Martínez, Mauro Dalla Serra, Mayra Tejuca

**Affiliations:** 1 National Research Council of Italy - Institute of Biophysics and Bruno Kessler Foundation, Trento, Italy; 2 Center for Protein Studies, Faculty of Biology, University of Havana, Vedado, Ciudad de La Habana, Cuba; CEA (Atomic and alternative energies commission), France

## Abstract

The use of pore-forming toxins in the construction of immunotoxins against tumour cells is an alternative for cancer therapy. In this protein family one of the most potent toxins are the actinoporins, cytolysins from sea anemones. We work on the construction of tumour proteinase-activated immunotoxins using sticholysin I (StI), an actinoporin isolated from the sea anemone *Stichodactyla helianthus*. To accomplish this objective, recombinant StI (StIr) with a mutation in the membrane binding region has been employed. In this work, it was evaluated the impact of mutating tryptophan 111 to cysteine on the toxin pore forming capability. StI W111C is still able to permeabilize erythrocytes and liposomes, but at ten-fold higher concentration than StI. This is due to its lower affinity for the membrane, which corroborates the importance of residue 111 for the binding of actinoporins to the lipid bilayer. In agreement, other functional characteristics not directly associated to the binding, are essentially the same for both variants, that is, pores have oligomeric structures with similar radii, conductance, cation-selectivity, and instantaneous current-voltage behavior. In addition, this work provides experimental evidence sustaining the toroidal protein-lipid actinoporins lytic structures, since the toxins provoke the trans-bilayer movement (flip–flop) of a pyrene-labeled analogue of phosphatidylcholine in liposomes, indicating the existence of continuity between the outer and the inner membrane leaflet. Finally, our planar lipid membranes results have also contributed to a better understanding of the actinoporin’s pore assembly mechanism. After the toxin binding and the N-terminal insertion in the lipid membrane, the pore assembly occurs by passing through different transient sub-conductance states. These states, usually 3 or 4, are due to the successive incorporation of N-terminal α-helices and lipid heads to the growing pores until a stable toroidal oligomeric structure is formed, which is mainly tetrameric.

## Introduction

Sticholysins (Sts) I and II (StI/II) are two very similar pore-forming toxins (PFT) isolated from the sea anemone *Stichodactyla helianthus*. They belong to actinoporins, a family of extremely potent PFT expressed by anemones. It is thought that these soft-bodied sedentary animals rely on their venomous secretions - among which actinoporins are important components - not only to paralyse and prepare prey for digestion, but also to defend themselves from predators [1], [2]. In natural habitats, sea anemones may prey on small crustaceans and non-symbiotic fish [3]. Nevertheless, the true biological role of actinoporins is still inconclusive [4].

The most studied members of this family are Sts and equinatoxin II (EqtII) from *Actinia equina*. Actinoporins are 20 kDa cysteineless proteins with preference for sphingomyelin-containing membranes. They are composed of a tightly folded β-sandwich flanked by two α-helices [5]–[9]; the N-terminal helix being involved in the transmembrane pore formation [10]–[13]. Other well studied protein domains involved in the initial membrane binding step are the exposed aromatic amino acid cluster situated on the broad loop at the bottom of the molecule and the C-terminal helix [14]–[18], and the phosphorylcholine (POC) binding site [5], [17]. The participation of an array of basic amino acids in the initial membrane recognition step has been also proposed [7]. However, the analysis of the actinoporins interfacial binding site predicts that electrostatic interactions do not play an important role in the membrane interaction [19].

Actinoporins constitute an attractive target in the construction of membrane-acting immunotoxins (ITs) directed against tumour cells. StI has been used to obtain these hybrid molecules with encouraging results [20]. With the purpose to improve such ITs specificity, StI W111C, a cysteine mutant of StIr in the membrane binding region was obtained [21]. In the proposed ITs, the mutant and the ligand moieties would be linked by proteinase-sensitive peptides through the StI W111C cysteine residue, blocking the toxin binding region and hence IT non-specific killing activity. The construction, purification and evaluation of proteinase-activated ITs, using StI W111C as toxic moiety, are in development at the moment. In the effort to better understand the IT-cell membrane interactions, we decided to carry out the functional characterization of this mutant considering that tryptophan 111 has been clearly identified, in a StIr NMR study, as one of the residues of the aromatic cluster that effectively contacts the membrane in the very early steps of pore formation [18]. Moreover, its implication in the EqtII interactions with micelles has also been demonstrated by NMR studies [22]. This is considered one of the two most important residues for sphingomyelin (SM) recognition by actinoporins [19]. However, no mutant of this residue has been studied in order to evaluate the impact of its substitution in Sts pore-forming capacity and, in this way, to corroborate its importance in the Sts lytic mechanism.

The actinoporins pore-forming mechanism recently proposed is characterized by three subsequent steps. The first is protein-membrane association mediated by exposed aromatic amino acids. The second is the insertion of N-terminal α-helices in the membrane in a non-coordinated way, and the third is the oligomerization process [13]. The oligomeric nature of actinoporin pores has been inferred from cross-linking experiments [23], kinetic data [24], electron microscopy analyses [5] and photobleatching approaches [25]. However, Rojko *et al.* (2013) demonstrated that the N-terminal region needs to be inserted in the lipid membrane before the oligomerization into the final pore occurs. This non-coordinated N-terminal α-helix insertion implies that actinoporins pore formation does not require a stable non-conductive prepore, preceding the concerted insertion of the oligomeric channels often found in other PFT. In fact, this is the most notable characteristic of the model recently proposed.

According to the protein structure inserted in the membrane, PFT are classified as α-PFT and β-PFT [26]. Actinoporins belong to the first group. It has been demonstrated that β-PFT often form β-barrel pores via a stable prepore. These structures allow the formation of hydrogen bonds between adjacent β-strands and in this way they guarantee the stability of the β-barrel once they are inserted in the lipid membrane [27]. In contrast, the formation of prepores has been shown only for two α-PFTs: the cytolysin A from *Escherichia coli* and *Salmonella enterica* [28], and the actinoporin fragaceatoxin (FraC) from the sea anemone *Actinia fragacea* [8]. In both cases the formation of an α-barrel-staves pore has been proposed. This type of pore is characterized by a very-well defined annular protein pore structure from which the lipids are excluded.

On the contrary to the cryoelectron microscopy studies of FraC that revealed the α-barrel-stave pore structure, other experimental evidences support the hypothesis that actinoporins form toroidal pores in which polypeptide chains and polar phospholipid headgroups are both involved [29], [30].

In the present work, we have confirmed the importance of the residue 111 in StI-membrane binding. In fact, the substitution of tryptophan by cysteine reduced the affinity of the toxin for the membranes, as demonstrated in experiments carried out in monolayers. This substitution impacted negatively to the activity of the mutant in erythrocytes, liposomes and planar lipid membranes (PLM). In the first two model systems, the mutant needed 10-fold higher concentration than the wild-type to reach the same effect; while in the histogram of pore conductances registered in PLM, the mutant StI W111C showed an additional population of low conductance pores less represented in the StIr histogram. In addition, we demonstrate in this paper that StIr and StI W111C form toroidal pores, in which the pore lumen is constituted by both, peptides and lipid headgroups, and where the lipids flip–flop is facilitated.

Notably, in this study, we observed that each pore formed by StIr or StI W111C is constituted by 3 or 4 short living sub-levels. These sub-levels are transient states toward the opening of the stable and bigger pore and may therefore support the independent and subsequent incorporation of 3 or 4 N-terminal regions, which finally constitute the final pore structure [5], [23], [24]. This observation completes the current model of the actinoporin pore-forming mechanism sustaining that each N-terminal region is inserted in the lipid membrane in a non-coordinated way before the oligomerization [13].

## Materials and Methods

### Ethics Statement

All procedures related to the use of human blood in this study, i.e. the Haemolytic Assay and the Pore Size Determination test, were carried out in conformity with the recommendation provided in The Code of Ethics of the World Medical Association (Declaration of Helsinki) for experiments involving humans in the Institute of Foods and Pharmacy, University of Havana (Cuba). The protocols were approved before the study began by the Ethics Committee of the Institute of Foods and Pharmacy, University of Havana, which is the Institutional Review Board of this University. The subjects provided verbal consent, following the explanation of the blood sampling procedure and the protocols briefly; a written consent was not seen necessary by the IRB and Ethics Committee as the procedure was accepted not to involve more than minimal risk. The information related to the volunteers are kept in a separate data file, which is not directly linked to the experimental data files.

### Toxins and Reagents

Recombinant wild-type *Stichodactyla helianthus* sticholysin I (StIr) and its mutant StI W111C were isolated as described [21], [31]. *S. aureus* alpha-hemolysin (αHL) was from Sigma, and gamma-hemolysin (HlgA/HlgB) was a kind gift of G. Prévost.

Calcein was obtained through Sigma Chemical Co. and Triton X-100 from Merck. Lipids Egg phosphatidylcholine (PC), 1,2-Dioleoyl-sn-Glycero-3-Phosphocholine (DOPC), 1,2 diphytanoyl-*sn*-glycerophosphocholine (DPhPC), porcine brain *sphingomyelin* (SM) and egg SM were from Avanti Polar Lipids (Alabaster). The 1-lauroyl-2-(1′pyrenebutyroyl)-*sn*-glycero-3-phosphocholine (pyPC) was kindly donated by Dr. P. Muller. Ethylene glycol (EG) was from Sigma. Polyethylene glycol 400 (PEG 400) was from Jansen and PEG200, PEG600 and PEG 1000 were from Fluka.

### Electrophoretic Analysis

SDS-PAGE. Denaturing gel electrophoresis was performed according to Laemmli [32], using 12.5% polyacrylamide gels. Gels were stained with Coomassie brilliant blue.

### Haemolytic Assay

Haemolytic activity was determined on human red blood cells (HRBC) by measuring changes in the attenuance at 600 nm with a 96-well microplate reader Multiskan “EX” (Labsystems, Finland). Briefly, HRBC were prepared from freshly heparinized blood, collected intravenously from healthy volunteers, by washing three times in 0.85% NaCl. Toxins were two-fold serially diluted in 100 µl of 140 mM NaCl, 10 mM Tris-HCl, pH 7.4 (TBS) starting from an initial concentration of 2.4 nM. 100 µl of HRBC in TBS were then added at an initial absorbance of 0.1 in the total volume. The toxin concentrations at which 50% of HRBC are lysed after 30 min (HC_50_) were estimated by the dose-effect sigmoidal fit plot using the OriginPro 7 software (OriginLab Corporation, Northampton, MA).

### Pore Size Determination

Pore size was determined in HRBC prepared as described above. Briefly, in each well, a fixed concentration of the toxins (1, 2 and 4 nM for StIr and 10, 20 and 40 nM for StI W111C) was present, in a final volume of 100 µl of TBS, with or without 30 mM of EG or PEG (PEG 200, PEG 400, Peg 600 and PEG 1000). The kinetic assay was started by adding to each well 100 µl of HRBC (preequilibrated with the same osmoticant), at a titre corresponding to an initial A_650_ value of around 0.1. The microplate was shaken and read every 12 sec for 1 h 30 min.

Addition of osmoticants, as EG and PEGs, increased the half-time of toxins hemolysis (t_½_), in a size-dependent manner. The difference t_½_ – t_½_°, the half-times in presence and absence of osmotic protectants, respectively, was used as an estimate of the introduced delay; this parameter measures the time necessary for the osmolite to diffuse inside the cell through the toxin-induced lesions. Accordingly, 1/(t_½_−t_½_°) is an estimate of the permeability of osmoticants through the pore, which was used to build a Renkin plot [33], [34], that provided an estimate of the pore radius. PEGs hydrodynamic radii were taken from [35], [36].

In all the experiments carried out using erythrocytes as membrane model, StI W111C was preincubated with 0.1 M 2-mercaptoethanol (2-ME) during 15 min before the assay.

### Permeabilization assay

Large unilamellar lipid vesicles (LUV) of PC and SM (1∶1) were used to check the ability of StIr and StI W111C to form active pores in model membranes. LUVs loaded with 80 mM calcein (a self-quenching condition) were obtained by extrusion through two stacked polycarbonate filters with 100 nm pores. The nonentrapped dye was removed by gel filtration on a microcolumn loaded with Sephadex G-50 gel, pre-equilibrated with 100 mM NaCl, 10 mM Tris-HCl, 1 mM EDTA, pH 7.4 (liposome buffer). StIr (0.1 µM) and StI W111C (2.4 µM) were 2-fold serially diluted in the same buffer used for gel filtration. The mixing of the vesicles with the toxins produced an increase in the calcein fluorescence F (due to the dequenching of the dye upon dilution into the external medium), which was measured using a fluorescence microplate reader (Fluostar; BMG LABTECH, Offenburg, Germany). Lipid concentration was 10 µM in all the experiments. The percentage of calcein release, R_%_, was calculated as follows:
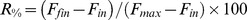
where F_in_ and F_fin_ represent the initial and the final (after 45 min) values of fluorescence before and after toxin addition, respectively. F_max_ represents the maximum signal obtained after the addition of 1 mM Triton X-100. C_50_ is the toxin concentration able to cause 50% of calcein release.

### Measurement of the transbilayer lipid movement

LUVs made of egg PC and porcine brain SM (1∶1) without calcein were prepared as described above in liposome buffer. The vesicles were then labeled on the outer leaflet as described by Muller *et al*. [37]. Briefly, an appropriate amount of pyPC dissolved in chloroform:methanol (1∶1) was evaporated under nitrogen and re-suspended in few µl of ethanol. 250 µM pyPC stock solution was prepared by adding liposome buffer. The asymmetrically labelled PC:SM liposomes were prepared by incubating for 20 min at 37°C the liposome suspension (20 µM final lipid concentration) with pyPC suspension 1 µM. The incorporation of pyPC into the outer membrane leaflet was followed in a photon counting fluorometer (SPEX FluoroMax, Horiba Jobin Yvon, Milan, Italy) using an excitation wavelength of 344 nm and measuring the fluorescence intensity of monomers (I_M_) and excimers (I_E_) at 395 and 465 nm, respectively. After the incorporation of pyPC into the outer leaflet of the vesicles, the resultant I_E_/I_M_ was set to 1. The transbilayer lipid movement after the addition of toxins was continuously followed by measuring the ratio I_E_/I_M_. If the toxin is able to form toroidal pores, a redistribution of the fluorescent lipid between the two leaflets is allowed and a decrease into the I_E_/I_M_ ratio should be measured. The protein concentrations in the assays were 500, 100, 50 and 10 nM for StIr and 600, 300 and 100 nM for StI W111C. In all the experiments based in the liposome model, StI W111C was treated with 10 mM dithiothreitol (DTT) before the assay.

### Surface Pressure Measurements in Monolayers

Surface pressure measurements were carried out by the Wilhelmy method, using two different setups, a KSV Minitrough Instrument and a *µTrough*-*S system from Kibron* (both from Helsinki, Finland) with circular Teflon troughs. The aqueous subphase volumes of the KSV and the Kibron *intruments* were 10 and 0.3 ml, respectively; the buffer used was 145 mM NaCl, 10 mM TrisHCl, pH 7.4. Lipids (DOPC and brain porcine *SM 1∶1)* dissolved in chloroform:methanol (2∶1) were gently spread over the subphase. Changing the amount of lipid applied to the water-air interface attained the desired initial surface pressure (π_0_). After approximately 20 min to allow solvent evaporation, StIr or StI W111C (50 or 1000 nM) were injected through a hole connected to the subphase. The increment in surface pressure versus time was recorded until a stable signal was obtained. All the measurements were taken at room temperature.

Prior to the injection, StI W111C was incubated with 0.1 M 2-ME for 15 min. Only in one experiment (see the Results session) the mutant was not preincubated with 2-ME. In that case, the reducer agent was added to the aqueous subphase 30 min after the toxin injection.

### Conductance Experiments in Planar Lipid Bilayers

Electrical properties were measured on PLM as reported by Dalla Serra and Menestrina [38]. Experiments were done at room temperature on solvent-free bilayers composed by DPhPC and egg SM in a 4∶1 molar ratio. Membranes were bathed symmetrically by 2 mL of PLM buffer (100 mM KCl, 5–10 mM Hepes, pH 7.0). Toxin (StIr or StI W111C) was added at nanomolar concentration (typically, 0.5–30 nM) on the *cis* side to stable preformed membranes with typical capacitance of 120–135 pF. A constant voltage of +40 mV was applied across the membrane at the *cis* side (the *trans* side was grounded). The current across the bilayer was measured, and the conductance (G) was determined as follows:
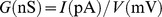
where *I* is the ionic current through the membrane, and *V* is the applied transmembrane electrical potential.

Macroscopic currents were measured with a patch-clamp amplifier (Axopatch 200, Axon Instruments, Union City, CA). A personal computer equipped with a DigiData 1200 A/D converter (Axon Instruments) was used for data acquisition. The current traces were filtered at 0.5 kHz and directly acquired at 2 kHz by a computer using Axoscope 8 software (Axon Instruments). For ionic selectivity determination, reverse voltages were measured in a stepwise increasing KCl gradient (with a final 10 times higher salt concentration in the *trans* side) and translated into a permeability ratio, P^+^/P^−^ (where P^+^ and P^−^ refer to cation and anion permeability, respectively), by the Goldmann-Hodgkin-Katz [38]. For these estimations, the pertinent activity coefficients for the *cis* and *trans* solutions were used.

For PLM experiments, both toxins (StIr and StI W111C) were preincubated with 0.1 M 2-ME for 15 min. Previous to the assays, the reducer agent was removed by desalting on Zeba Micro Spin Desalting Columns (from Thermo Scientific Pierce products).

## Results

### Hemolytic activity and pore radius estimation

**Figure 1:**
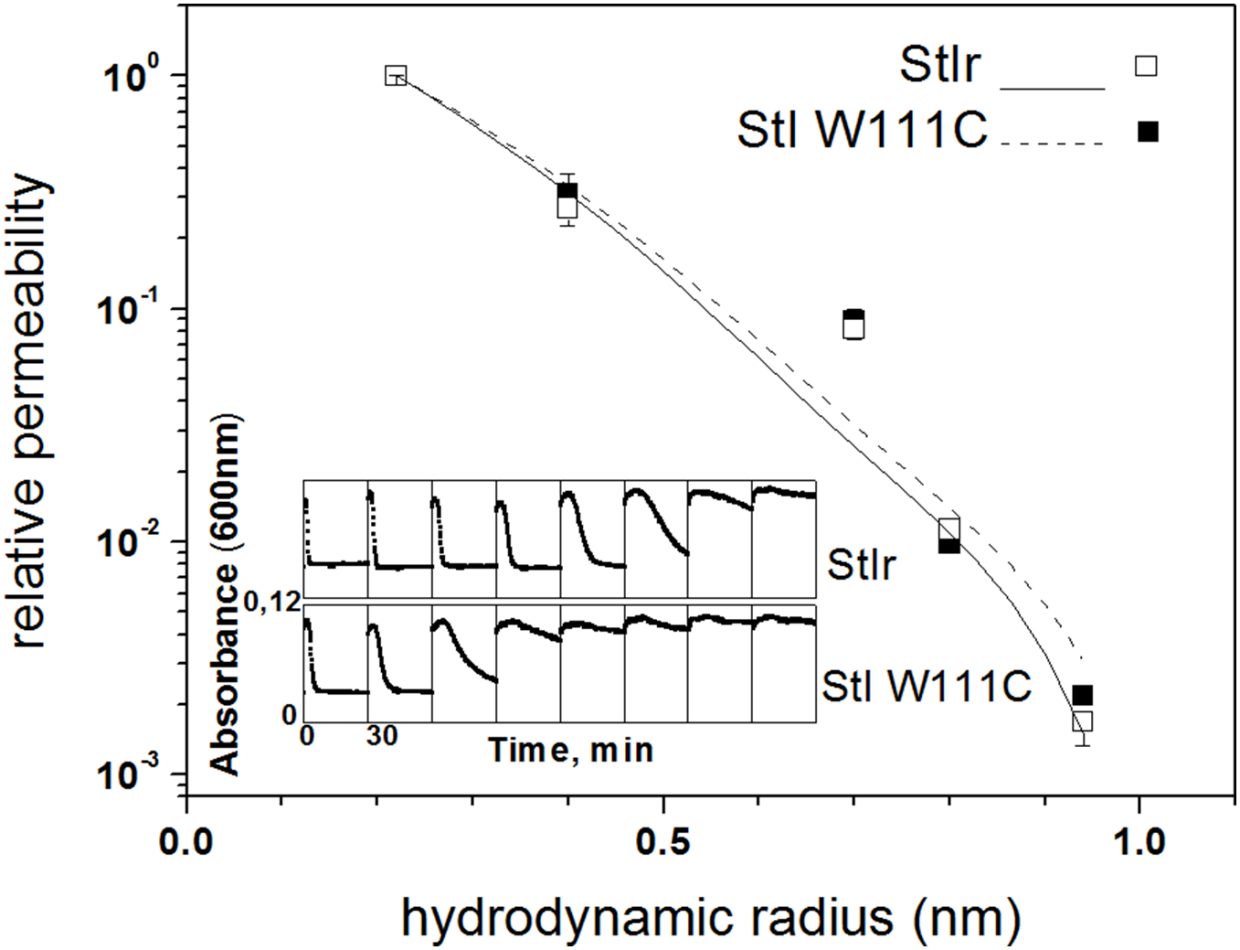
Haemolytic activity and radii estimation of pores formed by StIr and StI W111C in HRBC. Inset: The haemolytic activity of StIr (upper row) and StI W111C treated with 0.1 M 2-ME (lower row) were determined measuring changes in the attenuance at 600 nm of a HRBC suspension exposed to various doses of the toxin in a microplate reader. The protein concentration in the first wells (those on the left) was 1.2 nM. Toxins were diluted 2-fold in every next well, and eight different dilutions are shown in both cases. The axis scales are the same for all the wells and are reported only in the graph on the left bottom. The HC_50_ (concentration at which 50% of HRBC are lysed after 30 min) were 26 and 210 pM for StIr and StI W111C, respectively. The radii of pores formed by StIr and StI W111C treated with 0.1 M 2-ME were estimated by the Renkin plot. The rate of toxin-induced hemolysis promoted by three toxin concentrations (0.5, 1 and 2 nM for StIr and 5, 10 and 20 nM for StI W111C) were measured in the presence and absence of 30 mM final concentration of EG (0.22 nm); PEG 200 (0.4 nm); PEG 400 (0.7 nm); PEG 600 (0.8 nm) and PEG 1000 (0.94 nm). The number in parenthesis represents their hydrated radius in nm. An estimate of the permeabilities of EG and PEGs was derived from the inverse of the delay that they introduced in the hemolysis curves. Relative permeabilities of each PEGs were then obtained dividing these values by the permeability of EG. Reported values are the average ± SD of the permeability obtained in the three experiments carried out with each toxin, and the data were arranged in a Renkin plot providing an estimate of the pores radii. Solid and dashed lines are best fit of the Renkin for StIr (open squares) and StI W111C (closed squares), respectively. The radii estimated were 1.0±0.1 and 1.1±0.1 nm for StIr and StI W111C, respectively.

A titration of the hemolytic activity of StIr and StI W111C evaluated as described earlier [39] is shown in the inset of figure 1. The StI W111C HC_50_ is 210 pM, value around 10 times higher than that obtained for StIr (26 pM). Both values are in agreement with the HC_50_ previously reported: 18±8 and 141±95 pM for StIr and StI W111C, respectively [21].

The method used to estimate the radii of the pores formed by StIr or StI W111C takes advantage on the ability of the PFT to induce colloid–osmotic hemolysis. The radii estimation is based on the changed osmotic protective effect of PEGs with different sizes. Three different protein concentrations were used for both toxins. Under these conditions, the absolute rate of hemolysis varied; however, the effect of PEGs on the relative permeability was independent of the toxin concentrations, as previously described [40]. The lines in figure 1 are the best fits of the Renkin to the averages of the relative permeabilities obtained for the three toxin concentrations used in each case. These fits provided an estimate of the pore radii of 1.0±0.1 nm and 1.1±0.1 nm for StIr and StI W111C, respectively. These values are in agreement with that of native StI (i.e. 0.96 nm) as previously obtained [40].

### Permeabilization of Unilamellar lipid vesicles

**Figure 2:**
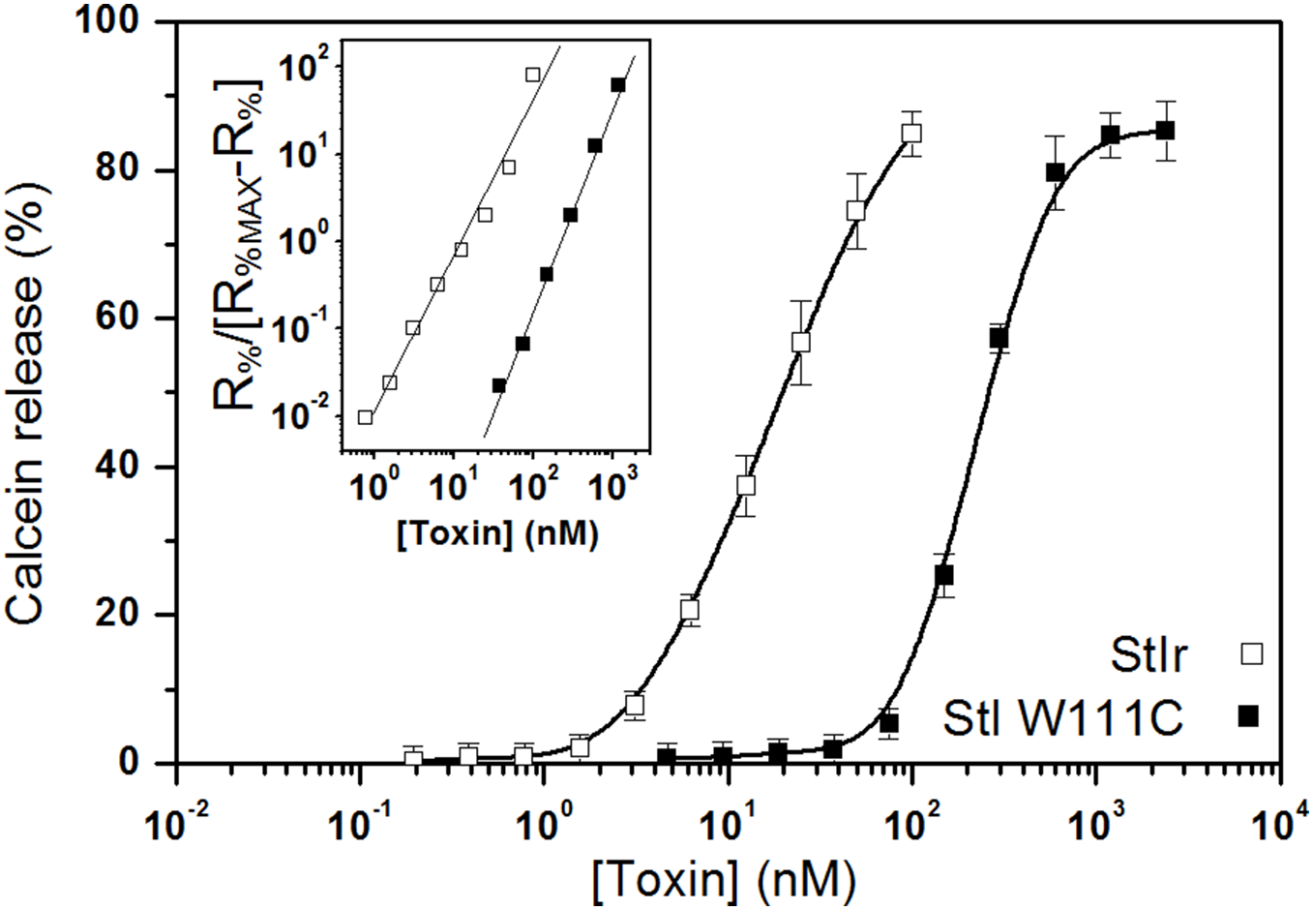
Dose dependence of LUV permeabilization induced by StIr and StI W111C. The capability of StIr (open squares) and 10 mM DTT treated StI W111C (closed squares) to form active pores in model membranes was measured using egg PC and porcine brain SM (1∶1) LUVs loaded with calcein (80 mM). The increase of fluorescence due to the action of different toxin concentrations was measured and the percentage of calcein release (R_%_), ± SD of two independent experiments, was estimated. Toxins were 2-fold serially diluted starting from maximal concentrations of 0.1 and 2.4 µM for StIr and StI W111C, respectively. Lipid concentration (10 µM) was constant in all the experiments. C_50_ (i.e., the concentration at which 50% of liposomes were permeabilized) were 20 and 212 nM for StIr and StI W111C, respectively. Inset: Hill plot. In order to apply the Hill approach to the permeabilization results, the StIr and StI W111C maximal % of calcein released (R_%MAX_) were estimated for interpolation in the dose dependence graphics. They were 85 and 86% for StIr and StI W111C, respectively. The linear segment of the plots for StIr (open squares) and 10 mM DTT treated StI W111C (closed squares) are shown in the inset. Solid lines are the best fits of the Hill equation, providing Hill coefficients of 1.7±0.1 and 2.3±0.1 for StIr and StI W111C, respectively.

Lipid vesicles are widely used as model systems to investigate actinoporins mode of action. A general characteristic of these toxins is their preference for SM containing membranes. In fact, vesicles formed by equimolar mixtures of SM and PC are very good targets for this toxins [24]. The dose-dependence of the toxins permeabilization was studied on calcein-loaded PC:SM (1∶1) LUV. In this model, StIr was again more active than StI W111C (figure 2), showing C_50_ of 20 and 212 nM for StIr and StI W111C, respectively. Nevertheless, in both cases the released calcein described a similar sigmoidal dependence on the toxin concentration, as was demonstrated by the Hill coefficients of 1.7±0.1 and 2.3±0.1 for StIr and StI W111C, respectively (figure 2, inset). The resemblance of these Hill coefficients suggests similarity in the molecularity of StIr and StI W111C oligomeric channels.

### Induction of lipid flip-flop in unilamellar lipid vesicles

Next, we investigated if the formation of StI W111C pores promotes the lipid flip-flop previously demonstrated for native StI [29]. In this case, we measured the transbilayer movement of a pyrene-labeled analogue of PC (pyPC), in PC:SM (1∶1) LUVs. This analogue of PC changes its fluorescence properties upon its redistribution between the membrane leaflets [37], [41].

**Figure 3:**
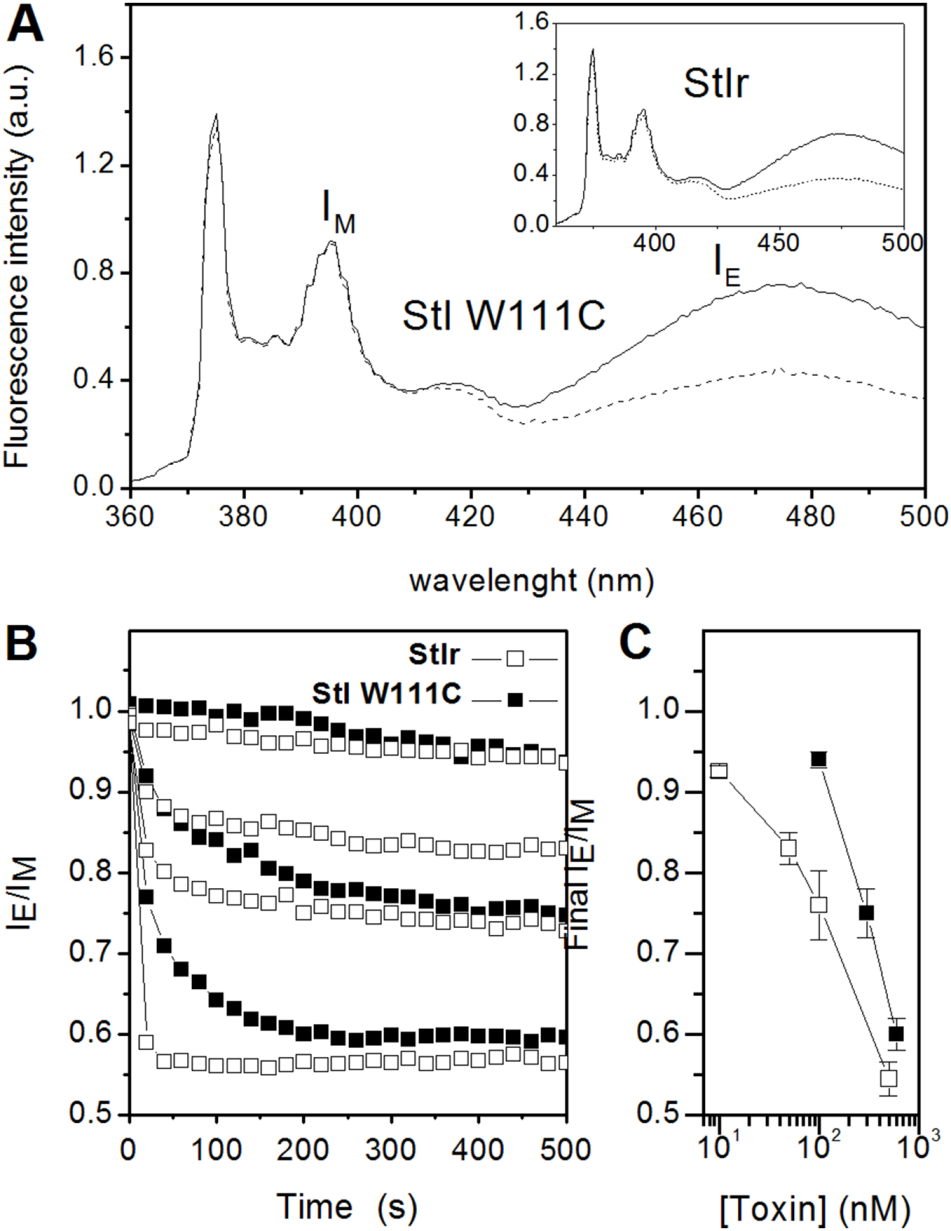
Transbilayer movement in presence of StIr and StI W111C. (A) Influence of StIr and StI W111C on the fluorescence spectra of pyPC in LUVs. Vesicles were made of egg PC:porcine brain SM (1∶1) labeled with pyPC at 5 mol% and the fluorescence spectra were recorded at 37°C using an excitation wavelength of 344 nm. The figure shows the initial pyPC fluorescent spectra (solid line), before toxin addition. The dashed line spectrum has been acquired at the steady state after the addition of the toxins treated with 10 mM DTT. The toxins were StI W111C or StIr (inset) at a final concentration of 600 or 500 nM, respectively. (B) Dose dependence of the transbilayer redistribution of pyPC induced by StIr and StI W111C. The time course of the transbilayer redistribution of pyPC was followed by measuring the fluorescence intensity of monomers (I_M_) and excimers (I_E_) at 395 and 465 nm, respectively, and calculating the ratio I_E_/I_M_. The protein concentrations in the assays were 500, 100, 50 and 10 nM for StIr (open squares) and 600, 300 and 100 nM for StI W111C (closed squares). (C) transbilayer redistribution of pyPC versus toxin concentration of StIr and StI W111C. The ratio I_E_/I_M_ reached at the steady-state (final I_E_/I_M_) in the experiments shown in panel B is reported (± SD of two independent experiments).

The fluorescence spectrum of pyrene is characterized by two signals, one arising from excited monomer molecules and the other from excimer molecules. The extent of excimer formation depends on the collision frequency of pyrene-labeled analogues, which is determined by their concentration. In the case of a porating agent able to induce the formation of toroidal pores, the two membrane leaflets become in contact and the phospholipid flip–flop is facilitated. Therefore pyPC, initially present only in the vesicles outer membrane leaflet, can redistribute in both leaflets causing a change into its fluorescence spectra. Figure 3A shows pyPC spectra in LUV of egg PC:porcine brain SM before (solid line) and 15 min after the addition of 10 mM DTT treated StI W111C at a final concentration of 0.6 µM (dashed line). The addition of the toxin provoked a significant decrease of the excimer fluorescence intensities, a clear indication of toroidal pores formation. A similar result was obtained when the effect of 0.5 µM StIr was evaluated (figure 3A, inset). After pyPC partitioning into the LUV outer leaflet and in the absence of toxin, the I_E_/I_M_ signal did not significantly change in our experimental conditions (not shown), indicating the stability of the liposome system.

The time course of the transbilayer redistribution of pyPC induced by different concentrations of the toxins was followed through the measure of the I_E_/I_M_ ratio. StIr and StI W111C induced pyPC flip-flop in PC:SM vesicles at all the tested concentrations, as demonstrated by the decrease of the I_E_/I_M_ signal (figure 3B). StIr was again more efficient both in rate and extent of pyPC transbilayer redistribution between the two leaflets as shown in figure 3C. This result correlates with the higher permeabilizing activity of StIr previously observed (figure 2). On the other hand, the addition of the toxins to vesicles did not affect their sizes, as was revealed by dynamic light scattering measurements (data not shown), indicating that toxins did not provoke any aggregation/fusion or micellization of the vesicles, in our experimental conditions. We concluded that, similarly to what previously published for Sts [29], StIr and StI W111C induced lipid flip-flop is caused by the formation of supramolecular toxin-lipid complexes, also named toroidal pores, which connect the outer and inner layers facilitating the free diffusion of lipid molecules between the two leaflets.

### Toxin adsorption into lipid monolayers

**Figure 4:**
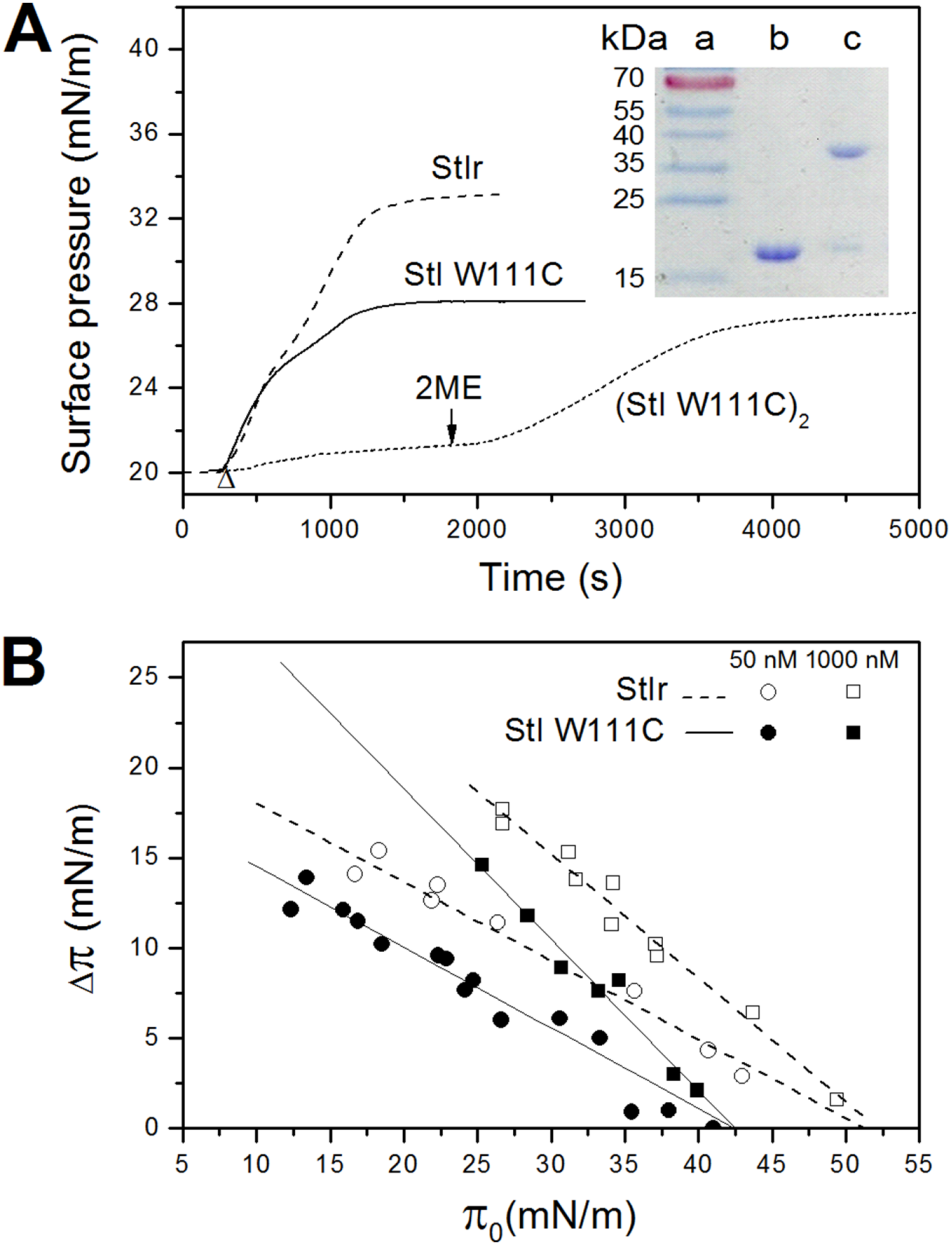
Insertion of StIr and StI W111C into lipid monolayers. (A) Adsorption isotherms of StIr and StI W111C into DOPC:brain porcine SM (1∶1) lipid monolayers at an initial surface pressure (π_0_) of 20 mN/m. The monolayers were prepared by adding lipids in small amounts until the desired π_0_ was reached. Once the signal stabilizes, the proteins were injected into the subphase (indicated by an arrow head). In the dotted line trace, labeled (StI W111C)_2_, StI W111C was added without previous treatment with 2-ME; consequently, the toxin was mainly in its dimeric form, as was demonstrated by SDS-PAGE (inset). The arrow indicates the addition of 0.1 M 2-ME ≈30 min after the toxin addition. The toxin concentration in the three experiments was 50 nM. Buffer composition was 145 mM NaCl, 10 mM TrisHCl, pH 7.4. Inset: SDS-PAGE analysis of the StI W111C sample. 5 µg of the toxin incubated with (lane b) and without (lane c) 2-ME were loaded on a 12.5% polyacrylamide gel. Lane a contains molecular weight markers (RageRuler Prestained Protein Ladder, Fermentas LIFE SCIENCES). Molecular weights are shown on the left. (B) Critical pressure plots. Increases in the surface pressure of the monolayers (Δπ) after the insertion of StIr (open symbols) or StI W111C (closed symbols) are plotted as a function of π_0_. The final protein concentrations were 50 (circle symbols) and 1000 nM (square symbols). The monolayer and the buffer composition are the same as in panel A. The critical pressures (π_c_), i.e. pressures at which no insertion can occur, were calculated from the linear fits of the experimental data shown in the figure. The values of π_c_ obtained for StIr at 50 and 1000 nM were 50.7 and 51.9 mN/N, respectively; while for StI W111C, the linear fits at the two protein concentrations data rendered approximately the same value of π_c_, 42.4 mN.

The ability of StI W111C to insert into lipid monolayers of the same lipid composition (DOPC:SM, 1∶1) used for permeabilization and flip-flop studies was also examined. The adsorption isotherms of StIr and StI W111C into monolayers with an initial lateral pressure (π_0_) around 20 mN/m are shown in figure 4A. StIr and reduced StI W111C added to the subphase were both able to induce an increase in the monolayer lateral pressure, StIr being the most effective as shown by the higher value of surface pressure reached at the steady state.

The high tendency of StI W111C to form dimers in solution has been already described in one of our previous papers [21]. In that work, the dimer was used to demonstrate that the binding of a bulky molecule to cysteine 111, e.g. an antibody, inactivates the mutant, inducing the loss of its ability to bind to the membrane, and therefore eliminating the IT non-specific activity. Since this is the main limitation of the ITs based on membrane-active toxins, the possibility to completely inactivate StI W111C mutant, makes it a good candidate for the construction of tumour proteinase-activated ITs.

In that previous work [21], we used erythrocytes and the hemolytic test to demonstrate the inactivation of (StI W111C)_2_. However, the inability of the dimer to bind to the membrane was more evident in the lipid monolayer model system where its insertion is directly visualized. In fact, when the mutant in not reductive condition was added to the monolayer, only a tiny increase of the surface pressure was observed (figure 4A trace (StI W111C)_2_). However, the addition of 2-ME 0.1 M to the subphase provoked an increase of the surface pressure. The steady state was reached at a value equivalent to that obtained by the mutant in reductive condition. The initial small increment of the surface pressure was due to the presence of a small quantity of the monomeric form in the sample, as confirmed by SDS-PAGE analysis (figure 4A, inset).

To compare StIr and StI W111C’s abilities to insert into lipid monolayers, we evaluated the increases of surface pressure (Δπ) at different initial pressure (π_0_). As expected, for both proteins at the two chosen concentrations, the Δπ decreased with the increase of π_0_ due to the tighter lipid packing that prevented the toxins insertion (figure 4B). The critical pressure (π_c_) at which no more protein inserts into the lipid monolayer was calculated from the linear fit as shown in figure 4B. The values obtained were independent of the protein concentration used in the experiments, and they reached around 51 and 42 mN/N, for StIr and StI W111C, respectively. This parameter is directly related to the protein affinity for the lipids in the monolayer, since it corresponds to the surface pressure that must be applied to avoid further toxin incorporation [42]. The higher value obtained for StIr clearly reveals that its insertion into the monolayer is easier than that of StI W111C. These results indicate that the tryptophan 111 substitution induces a decrease in the StIr lipid affinity confirming the previously reported importance of this residue for the actinoporins binding to the membranes [16], [18], [19], [22].

### Formation of Ionic Channels in Planar Lipid Bilayers

**Figure 5:**
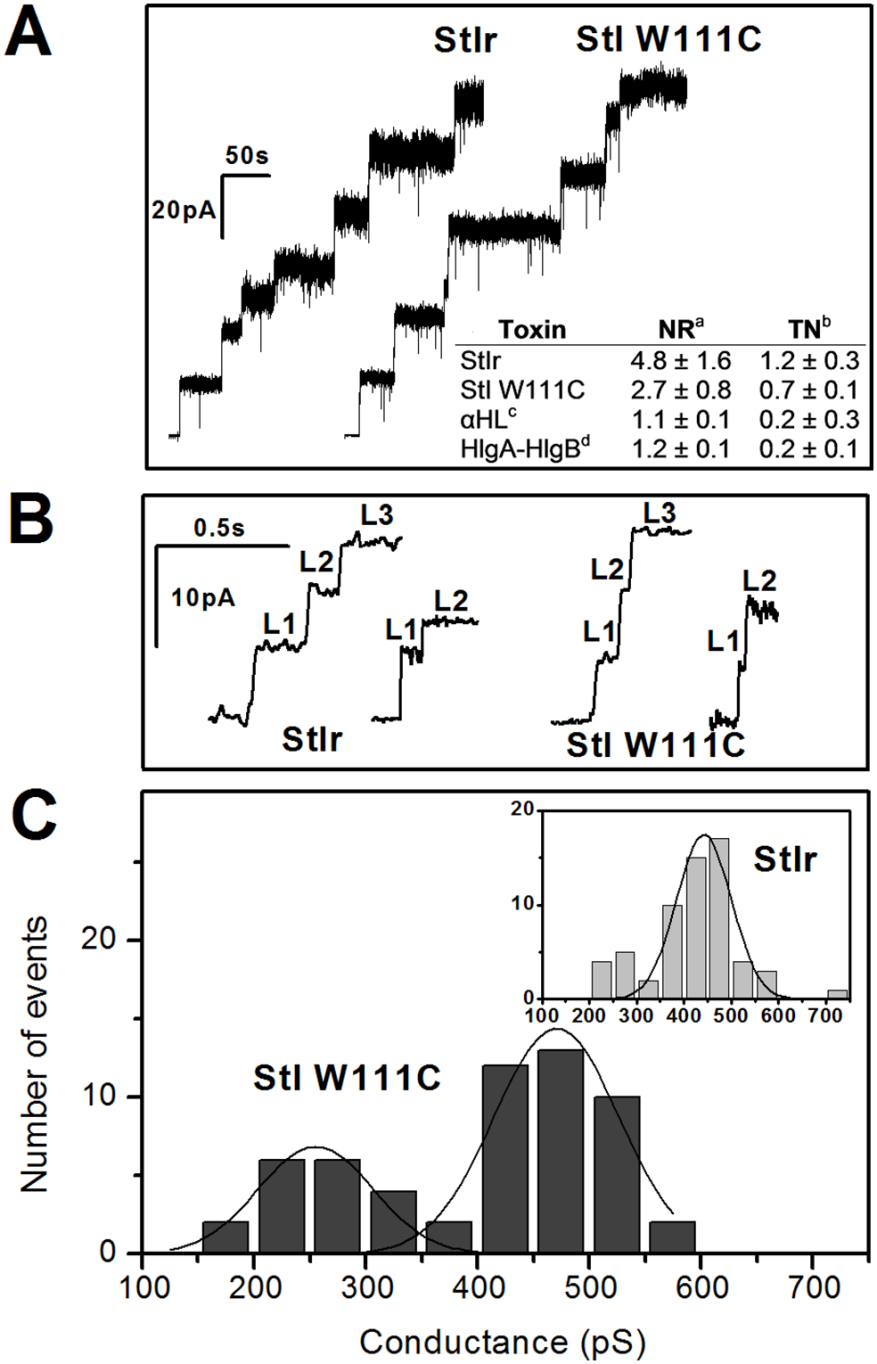
Pore-formation by StIr and StI W111C in PLM. (A) Addition of the toxins to one side of DPhPC:egg SM (4∶1) PLM with an applied voltage of +40 mV provoked the opening of ionic pores which increased the conductivity of the film in discrete steps starting from the zero current. Inset: Noise of the first open pore current level for some α- and β-PFT. Single pore currents were recorded at +40 mV; filtered at 500 Hz and acquired at 2 KHz. RMS noise was calculated from 30 s recordings of at least 3 independent experiments. Buffer salt concentration was KCl 100 mM. ^a^NR = NP/NB (± SD) where NP is the noise of the first open pore and NB is the noise of the intact bilayer. TN^b^ is the Toxin Noise = (NP^2^-NB^2^)^1/2^ (± SD). αHL^c^ and HlgA-HlgB^d^ correspond to *S. aureus* alpha- and gamma-hemolysin, respectively. (B) Representative traces of StIr and StI W111C time resolved consecutive sublevels. L1, L2 and L3 indicate three conductivity levels constituting stable fully structured pores. Their values for StIr traces are L1 = 7.0 pA, L2 = 12.6 pA and L3 = 17.9 pA; and L1 = 6.2 pA and L2 = 9.7 pA, for the left and right traces, respectively. For the StI W111C traces the values are L1 = 6.3 pA, L2 = 13.2 pA and L3 = 19.0 pA for the left trace and L1 = 5.42 pA and L2 = 11.19 pA for the right one. (C) Histogram of the StI W111C pore conductance. Fifty-seven events distributed in two peaks of average conductance (±SD) of 255±102 and 471±112 pS were cumulated. Inset: Histogram distribution of sixty-one StIr events; average conductance (±SD) was 442±116 pS. Other experimental conditions were as follows: both proteins were treated with 0.1 M 2-ME before these experiments; the toxin (StIr or StI W111C) concentration was 0.5–30 nM; and the bathing solution was 100 mM KCl, 5–10 mM Hepes, pH 7.0.

StIr and StI W111C pores were directly measured on PLM composed of PC:SM (4∶1) in 100 mM KCl pH 7.0. Both toxins caused stepwise increases in the conductivity of the film when clamped at fixed voltage, indicating the formation of pores (figure 5A). Pore openings cause also an increase in the noise level (figure 5A, inset). Interestingly, the opening of stable single pores occurred through 3 or 4 conductance sub-levels in rapid succession. Figure 5B shows representative traces of StIr and StI W111C with three resolved partial steps of 17.9 and 19.0 pA (i.e. 447 and 475 pS), respectively. Similar sub-levels have been previously observed also for native StI and StII (Tejuca M. Lytic mechanism of *Sticholysin I*, a cytolysin from the anemone *Stichodactyla helianthus*. Ph.D. Thesis, 1996, Universidad de La Habana, Cuba and G. Menestrina, personal communication). In addition, single pores formed by 2 sub-levels were also observed. Representative traces of StIr and StI W111C lower conductance channels of 9.7 and 11.2 pA, respectively (i.e. 241 and 280 pS) are shown in figure 5B. On the other hand, the analysis of the conductance histogram showed that, while most of the StIr pores belong to a single conductance peak with an average conductance around 440 pS (figure 5C, inset), the StI W111C pores were clustered into two clearly distinct peaks of average conductances around 260 and 470 pS (figure 5C).

The electrical properties of stable full pores formed by StIr and StI W111C in PLM were also investigated (table 1). The voltages at which no current flowed, in experiments where the membrane separated two solutions containing 100 mM KCl on the *cis* side and 1 M KCl on the *trans* side (reversal voltage, V_rev_), were estimated for both toxins. This parameter was very similar: 27.6 and 27.5 mV for StIr and StI W111C, respectively. From these analyses we estimated that both channels are cation selective, roughly 5 times more permeant to K^+^ than to Cl^−^ as indicated the ratio P^+^/P^−^ (table 1).

**Table 1:** Electrical properties of StIr and StI W111C channels in PLM.

	G (pS)^a^	V_rev_ (mV)^b^	P^+^/P^−*c*^	I^+^/I^−*d*^	r (nm)^e^
StIr	442±116	27.6±0.7	4.7±0.2	1.1±0.1	0.9±0.1
StI W111C	471±112	27.5±1.5	4.6±0.4	1.0±0.1	1.0±0.1

a G is the channel conductance, calculated as the average ± SD of the events reported in the histograms of figure 5C.

b V_rev_ is the voltage at which no current flows in an experiment in which the membrane separated two solutions containing 100 mM KCl on the *cis* side and 1 M KCl on the *trans* side.

c P^+^ and P^−^ represent the permeability of the cation and the anion respectively. The V_rev_ and P^+^/P^−^ are the averages of five and two independent determinations for StIr and StI W111C, respectively.

d I_+_ and I_-_ symbolize the current measured at +100 mV and −100 mV, respectively. In this case, at least three independent experiments have been performed and the I_+_/I_-_ error falls within 10%.

e r is the radius of the pore estimated from the channel conductance as described in the text.

The molecular properties of these pores were investigated in relation to the applied voltage. In both cases, instantaneous current-voltage characteristics (I–V) were found to be linear as indicate the ratios between the currents measured at 100 mV and −100 mV (I^+^/I^−^) around 1 (table 1).

In addition, StIr and StI W111C pore radii was estimated from their main pore conductances, as described by Tosatto *et al*. [43]. In these experiments, it was assumed that the channels are perfect cylinders 6 nm long. Considering that StIr and StI W111C conductances are 442 and 471 pS, respectively, and that 12 mS/cm is the solution conductivity (for 100 mM KCl solution), the radii estimated for StIr and StI W111C were 0.9±0.1 and 1.0±0.1 nm, respectively (table 1). These values are in agreement both with the pore sizes estimated by RBC assay, e.g. 1.0±0.1 and 1.1±0.1 nm for StIr and StI W111C, respectively (figure 1), and with the values previously estimated for native StI by PLM of 1.0±0.1 nm [24].

## Discussion

Tryptophan 111 is located in an aromatic rich region involved in the actinoporins anchoring to the membrane [14], [16], [22]. Specifically, in a NMR study carried out with StIr, this residue has been identified as one of the aromatic residues (together with the tyrosines 132, 136 and 137) that would be in intimate contact with the membrane in the very early steps of interaction [18]. On the other hand, there are evidences suggesting that in EqtII W112 and Y113 (equivalents to W111 and Y112 in StI) are responsible for the discrimination between SM and PC [19]. In fact, it has been proposed that actinoporin POC binding site with a bulky hydrophobic amino acid at position 112 and an aromatic ring at position 113 (numbers refer to the EqtII sequence), may represent a common 3D-structural motif able to bind a single SM molecule, either in solution or in a lipid bilayer [19].

In agreement with the importance of the tryptophan 111, StI W111C showed lower haemolytic and permeabilizing activity than StIr. In fact, the mutant’s HC_50_ and C_50_ were 10-fold higher than those obtained for the wild-type both in haemolysis (figure 1, inset) and calcein release (figure 2) analyses. The results obtained by monolayers experiments showed that the mutant lower activity is due to a decreased affinity to the membrane (figure 4B). In this membrane model system, the initial pressure required to prevent StIr incorporation into the monolayer was higher than the one needed by StI W111C, meaning that the wild-type is able to insert into more compact monolayers, supporting its higher affinity for membranes.

However, the fact that StI W111C conserves an important binding capacity is not surprising. Bakrac *et al.* [19] have pointed out that the presence of a bulky hydrophobic amino acid at this site plays a pivotal role. Cysteine is in the fifth position in the Kyte-Doolittle hydropathy scale [44], just after leucine and phenylalanine, aminoacids that have replaced this tryptophan in other actinoporins.

As part of the StI W111C functional characterization, we studied other pore features involved in the pore-forming activity. Using osmotic protectants, it was estimated that in human erythrocytes the radii of the pores formed by both toxins are around 1 nm (figure 1). These radii coincide with those previously estimated for native Sts and for EqtII using the same experimental model [23], [40] but also other approaches, such as radius estimation by the pore conductance in PLM [24], by the extent of the leakage and the molecular mass of vesicle entrapped solutes [45] and by osmometric behavior of permeabilized LUVs [40].

Hill coefficients obtained by the calcein release assays were around 2 (figure 2, inset), indicating the oligomeric nature of pores and suggesting a similarity between StIr and StI W111C channel molecularities. Based on kinetic data, it was inferred that native StI forms oligomeric pores comprising of at least three monomers [24]. Cross-linking experiments carried out with EtII indicated that the final pore has a tetrameric structure [23]. In agreement with this, electron microscopy analyses of StII two dimensional crystals on lipid monolayers revealed the presence of stable tetrameric pore-shaped structures [5]. Furthermore, recent photobleaching experiments support a heterogeneous distribution of EqtII pores, consistent with a tetrameric structure [25]. However, the notion that actinoporins channels are formed by three or four monomers was recently challenged by cryoelectron microscopy studies that showed a nonameric composition of the lipid-free α-helical bundle FraC pores [8]. This α-barrel stave structure contrasts with the actinoporins toroidal pore previously proposed by functional experimental evidences.

To this end, we asked ourselves if StI W111C pore promotes lipid flip-flop as previously demonstrated for native StI [29]. Also in these experiments StIr was more active than the mutant, and both toxins provoked pyPC flip–flop, evidencing the formation of functional toroidal protein–lipid pores (figure 3). In this architecture, the toxin pore-forming domain induces the lipid membrane to bend onto itself in a torus-like structure constituted by both peptides and lipid headgroups [46]. A further evidence in favor of this model was the marked increase of Sts permeabilizing activity induced by the presence of phosphatidic acid, strong inducer of negative membrane curvature, in liposomes [29]. The toroidal pore model has also been proposed for EqtII considering two main facts: 1) an isotropic component was observed in ^31^P NMR, which is consistent with the lipid disorder associated to such structures, 2) the increase of the pores cationic selectivity in the presence of negatively charged lipids [30]. The properties of the channels in PLM were also studied. In agreement with the toroidal arrangement, the pores formed in PLM by StIr and StI W111C showed an excess current noise (figure 5A), a typical characteristic of the toroidal channels. In the inserted table the noise increase caused by the first pore aperture for StIr and StI W111C is reported, as well as the behavior of two well known β-PFTs. This property was also clearly evidenced by Kristan *et al.* [4] through the comparison of the typical low noise barrel-stave pores formed by the staphylococcal haemolysins and the noisy EqtII toroidal channels. The difference between the barrel-stave and the toroidal pore ionic currents was recently demonstrated in a study carried out using mutants of the heptameric staphylococcal α-haemolysin pore, in which the transmembrane β-barrel was so severely truncated that it was unable to span the lipid bilayer; however, it was able to induce the formation of lipid pores. In contrast to the wild heptameric staphylococcal α-haemolysin pore, these mutant pores were characterized by an excess current noise due to their conformational flexibility, as a consequence of the lipidic nature of their walls in which the stabilizing β-barrel interstrand hydrogen bonds are absent [47].

On the other hand, the conductance histogram of StIr showed the typical broad distribution of actinoporin pores [4], [24] with an average conductance of 442 pS (figure 5C, inset), value consistent with the one previously estimated for native StI channels in PLM [24] and with the size of StIr pores obtained by human erythrocytes analysis (figure 1). Instead StI W111C conductance histogram showed two populations centered around 260 and 470 pS (figure 5C). In this case, the high conductance state approximately coincides with that of StIr. Anyway, the broad distribution observed in both cases indicates that the actinoporin channels have not the well-defined fixed stoichiometry that characterizes the barrel-stave pores [27]. Other electric characteristics of the pores, such as the absence of rectification and the cation selectivity, were similar for both toxins (table 1).

The present model of actinoporins pore-forming mechanism includes three sequential steps: (i) the initial membrane association mediated by exposed aromatic amino acids, (ii) the N-terminal region insertion in the membrane, (iii) and the oligomerization [13]. In this mechanism, the N-terminal region of each monomer is inserted in the lipid membrane before the oligomerization in a non-coordinated way. Sustaining this mechanism, the actinoporin N-terminal α-helix can exist at the lipid-water interface and in the lipid membrane core as single unit. In the inserted protein configuration, a stable hydrogen bond network is formed within the helix itself, therefore non destabilizing free hydrogens are exposed to the hydrophobic milieu of the lipid membrane [48], and the formation of stable prepores are not necessary.

**Figure 6:**
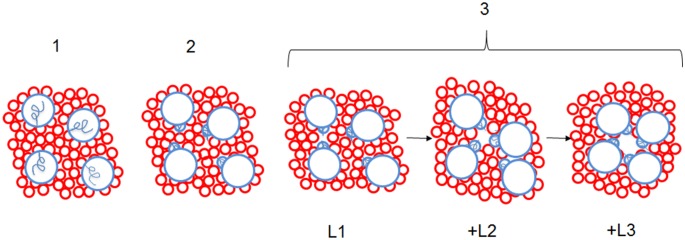
Cartoon representing the proposed mechanism of actinoporins pore assembly. Main steps leading to the formation of a tetrameric pore: (1) binding of toxin monomers to the membrane (2) insertion of the N-terminus into the membrane (3) oligomerization of toxin monomers via lateral diffusion on the membrane surface and generation of transient pores with increasing radii; the growing process ends when a final tetrameric pore is formed. L1, L2 and L3 refer to the conductance of each consecutive sublevel as shown in the inset of figure 5A. Toxins and inserted N-terminal α-helices are represented by big and small blue circles, respectively. The non-inserted N-terminal α-helices are represented in (1) as blue curls. Lipid heads are represented by red circles.

The current resolution of the PLM experimental model allows the detection and analysis of single-channel events. It was observed that the formation of StIr and StI W111C single pores frequently takes place through 3 or 4 conductance increases (sublevels or transient states) before reaching the more stable and bigger pore configuration, characterized by an average conductance around 440–470 pS (figure 5B). Such sublevels had been already observed in PLM studies of native StI pore formation (Tejuca M. Lytic mechanism of *sticholysin I*, a cytolysin from the anemone *Stichodactyla helianthus*. Ph.D. Thesis, 1996, Universidad de La Habana, Cuba) and StII (G. Menestrina, personal communication). However, in the traces of EqtII they are more rapid and not easily resolved (M. Dalla Serra, personal communication). Probably, the first increase in the films conductance could be the result of an appropriate contact via lateral diffusion of at least two toxin monomers. A suitable proximity of the two inserted N-terminal α-helices would originate a lower ion conductive pathway which corresponds to the first observed sub-level (L1 in the figure 5B). The next sub-level (L2 in the figure 5B) could be related to the incorporation of a further monomer to the toxin aggregate and so on until a final stable pore is reached. Most of the times it may be constituted by three or four toxin units with a final internal radius around 1 nm, as schematized in the cartoon (figure 6). This process could stop at any of the observed transient states, originating pores of smaller dimensions corresponding to lower conductance. Some single pores with lower conductance (240–280 pS) and formed by 2 sub-levels have been indeed observed. Representative traces of such channels for StIr and StI W111C are shown in the figure 5B. On the contrary, this process could continue and the pore would reach a final stable state with a bigger size. Similar results were obtained by cross-linking experiments that did not report the existence of EqtII oligomeric channels of single size, but rather various coexisting sizes up to, at least, the heptamer [23]. In agreement with these results, actinoporin pores are characterized by a broad conductance distribution (figure 5C). To this heterogeneity could contribute not only the number of N-terminal α-helices, but also the quantity of lipids forming part of the toroidal pore walls.

The low conductance peak in the StI W111C histogram could be due to the lower membrane affinity of the mutant and, consequently, lower effectively-bound protein concentration. If the bound toxin could be considered the limiting element in pore formation, the N-terminal insertion could stop at the second sublevel for many pores, resulting in the additional peak of lower conductance as observed for the mutant.

In summary, the results obtained in this work corroborate the importance of residue 111 for the binding of actinoporins to membranes. In addition, we have presented new evidences suggesting that actinoporins lytic structures assume toroidal protein-lipid pore structure. Concretely, the toxins provoke phosphatidylcholine transbilayer movement (flip-flop) in liposomes, indicating the existence of continuity between the outer and the inner membrane leaflet [4]. In this study we have shown some actinoporin channel characteristics that agree with the toroidal nature of the pore, i.e.: (i) the pores formed in PLM showed an excess current noise, due presumably to their conformational flexibility as consequence of the presence of lipids in the pore walls; and (ii) the channel conductance histograms exhibited a broad distribution, evidencing that pores do not have the well-defined fixed stoichiometry that characterizes the barrel-stave pores. Finally, the main novelty of this work is its contribution to the better understanding of the actinoporin’s pore assembly mechanism. Previously, it had been demonstrated that the insertion of the N-terminus into the membrane takes place in a non-coordinate way, shortly after the toxins binding and before the oligomerization into a final pore [13]. Our PLM results show that, during its structuration, each channel passes through different transient states, due probably to the incorporation of a growing number of monomeric α-helices -and lipids-, until it reaches a final stable oligomeric pore.

Additionally, from an experimental point of view, our results point out the system Sts-PLM as an ideal tool to study more accurately the non-coordinated N-terminal insertion of actinoporins.
